# Paeoniflorin Alleviates Lipopolysaccharide-Induced Neuroinflammation and Depression Through the Keap1/Nrf2/HO-1 Signaling Pathway

**DOI:** 10.3390/antiox14050585

**Published:** 2025-05-13

**Authors:** Zhuoyue Hu, Xing Wang, Tian Shi, Lei Yang, Boxi Zhang, Bo Shang, Ruizhi He, Shichen Yi, Jiao He, Jing Hu, Yanjun Cao

**Affiliations:** 1 School of Medicine, Northwest University, Xi’an 710069, China; 2022113236@stumail.nwu.edu.cn (Z.H.); hujinglzbx@163.com (J.H.); 2 Key Laboratory of Resource Biology and Biotechnology in Western China, Northwest University, Xi’an 710069, China; guairen825950330@sina.com (X.W.); 2023113137@stumail.nwu.edu.cn (T.S.); 2023113147@stumail.nwu.edu.cn (L.Y.); 2023113146@stumail.nwu.edu.cn (B.Z.); shangbo@stumail.nwu.edu.cn (B.S.); he_ruizhi2022@163.com (R.H.); 202233191@stumail.nwu.edu.cn (S.Y.); hejiao@nwu.edu.cn (J.H.)

**Keywords:** paeoniflorin, depression, lipopolysaccharide, neuroinflammation, Nrf2/HO-1

## Abstract

Depression is associated with bidirectional interactions between inflammatory responses and behavioral dysfunction. Paeoniflorin (PF), a monoterpene glycoside derived from *Paeonia lactiflora*, exhibits potent anti-inflammatory properties. This study investigates the therapeutic effects of PF on lipopolysaccharide (LPS)-induced depression-like behaviors in mice and neuroinflammation in BV2 microglial cells. Mice were co-administered PF (20, 40, or 80 mg/kg/day) and LPS (2 mg/kg) for 7 days. Behavioral tests; Nissl staining; and Golgi, Iba1, DLG4, and cytokine assays were conducted. Additionally, hippocampal NF-κB, Nrf2, and BDNF signaling pathways were analyzed using Western blots. In BV2 cells, oxidative stress and the Nrf2/HO-1 pathway were assessed using CCK-8, flow cytometry, and Western blotting after 24 h of LPS and PF treatment. PF significantly alleviated LPS-induced depression-like behaviors, increased hippocampal neuron and dendritic spine density, and upregulated synaptic proteins (PSD95, SNAP25, and BDNF). Mechanistically, PF suppressed NLRP3 inflammasome activation via the Akt/GSK3β pathway, reduced pro-inflammatory cytokines (TNF-α, IL-1β, and IL-6), and enhanced the Nrf2/HO-1 antioxidant axis. In BV2 cells, PF restored mitochondrial membrane potential, inhibited apoptosis, and decreased cytokine levels (TNF-α, IL-1β, and IL-6) by inhibiting TLR4/NF-κB signaling. In conclusion, PF significantly improved LPS-induced depression-like behaviors and attenuated neuroinflammation in BV2 microglial cells, highlighting its potential as a therapeutic agent for inflammation-associated depression.

## 1. Introduction

Depression and neuroinflammation exhibit a bidirectional relationship, where inflammatory cytokines exacerbate depressive symptoms [1]. Inflammation is a quick, dynamic bodily response triggered by stimuli. Inflammatory cytokines are key in this process and can cause depression-like behaviors [2]. Lipopolysaccharide (LPS), a potent inducer of systemic inflammation, triggers microglial activation, cytokine release, and hippocampal neurodegeneration, recapitulating key features of depression [3]. In mice, a peripheral injection of endotoxin LPS triggers brain microglial activation and perivascular macrophages to release cytokines, resulting in depression [4,5]. LPS activates TLR4 in microglia, triggering the release and activity of pro-inflammatory factors such as IL-1β and IL-6 and the generation of reactive oxygen species (ROS), leading to oxidative stress damage in the brain. Nrf2 is a key transcription factor in the cellular antioxidant defense system, regulating the expression of various antioxidant enzymes like SOD and GSH by activating the antioxidant response element (ARE). This process helps clear excess ROS and lipid peroxides such as MDA, activates the antioxidant defense system, inhibits neuroinflammation, and enhances neural plasticity, playing a crucial protective role in depression and serving as a potential therapeutic target [6]. Moreover, depressed mice have significantly lower Nrf2 levels in the hippocampal CA3 region compared to controls. Anti-inflammatory drugs effectively improve depression-like behaviors by reducing the levels of inflammatory cytokines and oxidase stress [6].

Autopsies of patients with depression revealed reduced densities of glial cells, pyramidal neurons, and granulosa cells across all hippocampal areas, along with significantly smaller pyramidal neurons [7]. NLRP3 is an apoptosis-related spotted protein found in human astrocytes, comprising a caspase-1 CARD (ASC) domain, a nitrogen (N)-terminal PYD domain, and a carbonyl (C)-terminal domain. Internal inflammation of neurons is beneficial for normal physiological processes, including brain plasticity, tissue repair, and neuroprotection. It protects them by eliminating or inhibiting various pathogens. However, over time, persistent neuroinflammation can lead to neuronal death or neurotoxicity. Excessive stimulation or prolonged stress can trigger inflammatory cytokines, leading to reduced neurotrophic factors, decreased neurogenesis, glial cell apoptosis, and cognitive dysfunction, all linked to depression [8]. Neurotrophic factors, particularly brain-derived neurotrophic factor (BDNF), play a crucial role in the long-term decline in neuronal plasticity associated with mental illness [8,9]. BDNF binds to its receptor TrkB, participates in neuronal development, and regulates synaptic plasticity. Inflammation can lower BDNF gene and protein expression levels and reduce TrkB expression, affecting neural plasticity. A peripheral injection of LPS can cause cognitive impairment and elevate hippocampal TNF-α and IL-1 levels, leading to reduced BDNF and TrkB expression and decreased hippocampal neurogenesis [4].

**Figure 1:**
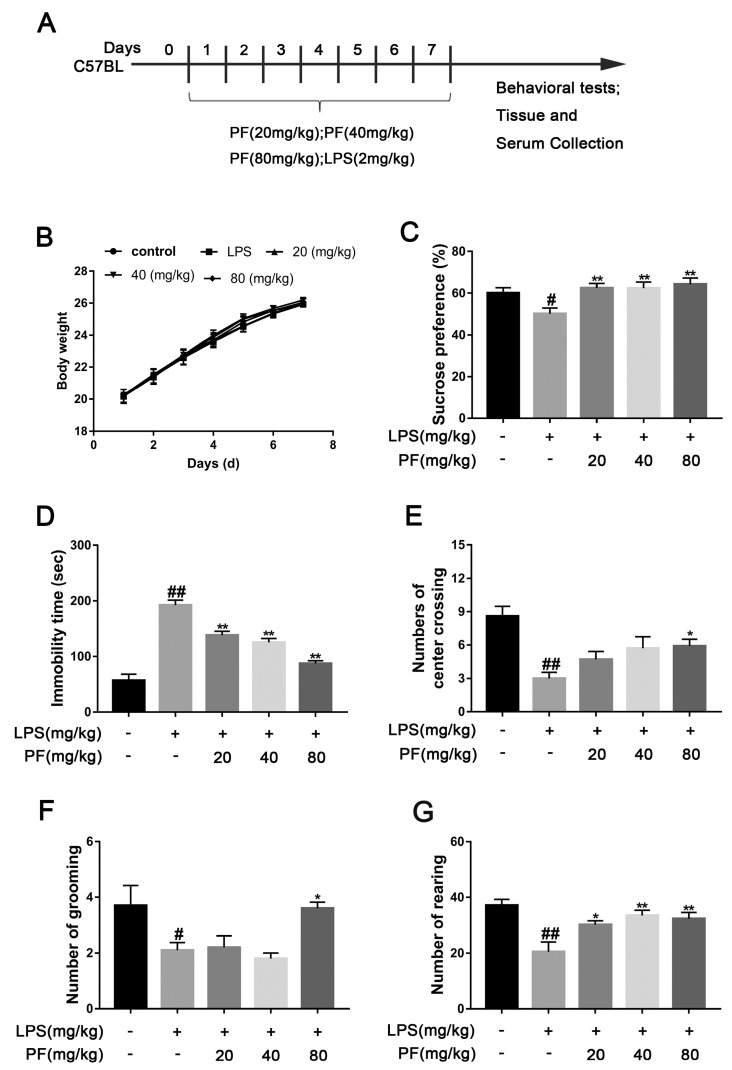
The impact of paeoniflorin (PF) on LPS-induced depression-like behavior in C57 mice. (**A**) Timeline showing a summary of the experimental design. (**B**) Body weight. (**C**) Sucrose preference in SPT. (**D**) Immobility time in FST. (**E**) Number of center crossings. (**F**) Number of grooming actions. (**G**) Number of rearing events. All data are presented as mean ± SEM (*n* = 10). * *p* < 0.05, ** *p* < 0.01, compared to the LPS group. ^#^ *p* < 0.05, ^##^ *p* < 0.01, compared to the control group.

Paeoniflorin (PF, Figure 1A) is a water-soluble monoterpenoid glycoside from the dried roots of *Paeonia lactiflora Pall*, a plant from the Ranunculaceae family. Our previous research found that PF played an antidepressant role in male offspring rats under prenatal stress, partly due to the reduction in HPA axis hyperfunction and improvements in glucocorticoid receptor dysfunction and the Glu transport system [10]. PF mitigated Glu-induced oxidative stress in SH-SY5Y cells through the Nrf2/HO-1 pathway and reduced cell apoptosis [11]. Research also has found that PF can inhibit the HMGB1/TLR4/NF-κB pathway to prevent depression induced by systemic lupus erythematosus [12]. However, its role in NLRP3 inflammasome regulation and synaptic plasticity during LPS-induced depression remains unexplored. This study integrates behavioral, histological, and molecular approaches to elucidate PF’s mechanisms in mitigating neuroinflammation and depression-like behaviors.

## 2. Materials and Methods

### 2.1. Animal Models and Grouping

Sixty male C57BL mice were randomly assigned to five groups (*n* = 12/group): control (saline), model (LPS 2 mg/kg), PF low dose (20 mg/kg/day and LPS 2 mg/kg), PF medium dose (40 mg/kg/day and LPS 2 mg/kg), and PF high dose (80 mg/kg/day and LPS 2 mg/kg), according to previous studies [11,12,13]. Each dose group of PF was given corresponding doses of PF by gavage, while the remaining groups were given 0.9% sodium chloride aqueous solution by gavage. One hour after PF administration, the model and PF dose groups received an intraperitoneal injection of 2 mg/kg LPS in a 0.2 mL volume. The control group was given 0.9% sodium chloride aqueous solution. Behavioral tests were performed after 7 consecutive days of administration.

### 2.2. Antibodies

Antibodies for WB included those against BDNF (A1307, ABclonal, Wuhan, China), TrkB (A12325, ABclonal, Wuhan, China), p-JNK (AP0631, ABclonal, Wuhan, China), JNK(A11119, ABclonal, Wuhan, China), p-ERK (A11119, ABclonal, Wuhan, China), ERK, p-P38 (AP0057, ABclonal, Wuhan, China), P38 (A10832, ABclonal, Wuhan, China), TLR4 (A11226, ABclonal, Wuhan, China), p-NF-κB (AP0123, ABclonal, Wuhan, China), NF-κB (A547, ABclonal, Wuhan, China), NLRP3 (A12694, ABclonal, Wuhan, China), Nrf2 (A1244, ABclonal, Wuhan, China), HO-1 (A19062-50, ABclonal, Wuhan, China), Keap-1 (A17061-50, ABclonal, Wuhan, China), NQO-1 (A19586-50, ABclonal, Wuhan, China), p-AKT (AP1208, ABclonal, Wuhan, China), AKT (A18675, ABclonal, Wuhan, China), p-GSK-3β (AP0039, ABclonal, Wuhan, China), GSK-3β (A3174, ABclonal, Wuhan, China), p-IκBα (AP0999, ABclonal, Wuhan, China), IκBα (A19174, ABclonal, Wuhan, China), COX (A1253, ABclonal, Wuhan, China), and iNOS (14124-1-AP, Sanying, Wuhan, China) as well as anti-mouse IgG (AS014, ABclonal, Wuhan, China) secondary antibodies.

### 2.3. Sucrose Preference Test (SPT)

Mice were housed individually with 50 mL of sucrose solution and 50 mL of purified water for 24 h, and bottle positions were switched every 12 h. During the experiment, after a 4-h fast, each cage received a bottle of 2% sucrose water and a bottle of water, with positions switched every 4 h. After 8 h, the bottles were removed, and consumption was recorded. The sucrose preference index was calculated using the following formula: sucrose preference index = (sucrose intake/total fluid intake) × 100).

### 2.4. Forced Swim Test (FST)

An open glass cylinder (30 cm high, 10 cm diameter) was filled with water to a depth of 25 cm at 25 ± 1 °C. Over a 6-min observation, the mice’s struggling and immobility times were recorded. Immobility is defined as the mouse being still or only slightly moving with one front paw. After the experiment, the mice were removed and dried, and the water in the circular cylinder was replaced for the next experiment.

### 2.5. Open Field Test (OFT)

The mice were placed in a 50 cm × 50 cm × 40 cm box with a 5 × 5 grid. After 5 min of observation, starting from the same position, the number of times each mouse crossed the center, stood vertically, and groomed were recorded. After each experiment, ethanol was sprayed in the box to prevent odor interference.

### 2.6. Cytokine Detection

Levels of the inflammatory cytokines TNF-α (JL10484, Jianglai, Shanghai, China), IL-1β (JL18442, Jianglai, Shanghai, China), IL-6 (JL20268, Jianglai, Shanghai, China), and IL-10 (JL18442, Jianglai, Shanghai, China) in serum were measured using an ELISA kit (Jianglai, Shanghai, China). The sample were added to a 96-well plate with a primary antibody. Then, 100 μL of antibodies was added per well followed by incubation at 37 °C for 1 h. Discard the liquid, wash the wells 5 times with detergent, and conduct a 15-min dark reaction with substrates at a constant temperature. Stop the reaction with a termination solution, and measure the OD at 450 nm.

Cells were seeded in a 6-well plate and cultured for 24 h. They were then co-incubated with LPS and varying concentrations of PF (10^−6^, 10^−5^, 10^−4^ M) for another 24 h. Afterward, cells were collected and centrifuged, and the supernatant was analyzed for the concentrations of the inflammatory cytokines TNF-α (JL10484, Jianglai, Shanghai, China), IL-1β (JL18442, Jianglai, Shanghai, China), IL-6 (JL20268, Jianglai, Shanghai, China), and IL-10 (JL18442, Jianglai, Shanghai, China) as per the ELISA kit instructions.

### 2.7. Nissl Staining

Animals were decapitated under 20% urethane anesthesia. Brains were extracted, paraffin-embedded, sliced into sections that were 4 μm thick, and stained with toluidine blue [13]. Nissl bodies in the hippocampal CA3 region were examined and photographed at 400× magnification using a light microscope. Neurons were counted in five evenly spaced coronal sections per brain using Image-J software (version 1.8.0 112, National Institutes of Health, Bethesda, MD, USA).

### 2.8. Staining with Golgi-Cox

The fixed brain tissue was rinsed gently with physiological saline several times. The brain tissue containing the hippocampus was cut, and it was stained with the Golgi-Cox staining solution. After 14 days of dark treatment, the tissue blocks were dehydrated. Then, they were treated with distilled water and concentrated ammonia water. After treatment, the tissue slices were reacted with a developer solution, dehydrated, and frozen. The slices were sealed with glycerol gelatin, and their images were obtained using a microscope.

### 2.9. Immunofluorescent Staining

Embed the whole brain in paraffin and cut into 4 μm sections. Perform antigen repair, wash with PBS, and block. Incubate with Iba1 (1∶500, Abclonal, A19714), NLRP3 (1∶500, Abclonal, A12694), and DLG4 (1∶500, Abclonal, A6194) primary antibodies. On the next day, add secondary antibody and diluted DAPI solution, rinse with running water, dry, apply anti-fluorescence quenching sealing agent, and observe under a fluorescence microscope. Image-J software (version 1.8.0 112, National Institutes of Health, Bethesda, MD, USA) was used to measure the regions in the hippocampus that were positive for Iba1, NLRP3, and DLG4.

### 2.10. Cell Culture and Treatments

BV2 microglial cells from Servicebio (Wuhan, China) were cultured in high-glucose Dulbecco’s Modified Eagle Medium (DMEM) from Gibco (Carlsbad, CA, USA), supplemented with 10% fetal bovine serum from Gibco (Carlsbad, CA, USA) and 1% penicillin (100 U/mL)/streptomycin (100 µg/mL) from BasalMedia (Shanghai, China). The cells were kept in a 37 °C environment with 5% CO_2_. For experiments, cells were treated with varying PF concentrations (10^−4^, 10^−5^, 10^−6^, 10^−7^, 10^−8^ M) and 1 μg/mL LPS for 24 h, followed by exposure to 5 mM ATP for the final 30 min.

### 2.11. Cell Viability Assay

BV2 cells were seeded in a 96-well plate at a density of 2 × 10^4^ cells per well and incubated for 24 h. They were then treated with varying concentrations of PF (10^−4^, 10^−5^, 10^−6^, 10^−7^, 10^−8^ M) and LPS (1 μg/mL) for 24 h, consistent with previous studies [14,15], followed by exposure to 5 mM ATP for the final 30 min. Following this, 10 μL of CCK-8 solution (Zeta Life, Shanghai, China) was added to each well, and samples were subject to a 1.5-h incubation period. Absorbance was measured at 450 nm.

### 2.12. ROS Detection with DCFH-DA Staining

We utilized a 2,7-dichlorodihydrofluorescein diacetate (DCFH-DA) fluorescent probe to measure intracellular reactive oxygen species (ROS) (Nanjing Jiancheng Bioengineering Institute, Nanjing, China). BV2 cells were co-incubated with LPS at 1 μg/mL and varying concentrations of PF (10^−6^, 10^−5^, 10^−4^ M) in a 6-well plate with 24 × 24 mm coverslips. The cells were stained with a 10 μM DCFH-DA solution for 30 min, followed by a 10 μM DAPI stain for another 30 min. The coverslips were then carefully removed using a needle from a syringe. The coverslips were sealed, dried in the dark, and examined under a laser confocal microscope (TCS SP8, Leica, Frankfurt, Germany).

### 2.13. Mitochondrial Membrane Potential Measurement

The JC-1 fluorescent probe (Beyotime, Shanghai, China) was used to measure Δψm in BV2 cells. Inoculate cells in a 6-well plate and then treat with LPS (1 μg/mL) and varying PF concentrations (10^−6^, 10^−5^, 10^−4^ M) for 24 h. The number of cells with low mitochondrial membrane potential was detected by flow cytometry after the cells were digested with trypsin, washed three times with PBS, and incubated with JC-1 fluorescent probe (5 μg/mL) for 30 min. The cells were collected, washed twice with PBS, and analyzed using flow cytometry (FACSAri3, BD Biosciences, USA).

### 2.14. Apoptosis Analysis Using Flow Cytometry

BV2 cells were treated with PF (10^−6^, 10^−5^, 10^−4^ M) and LPS (1 μg/mL) for 24 h, followed by exposure to 5 mM ATP for the final 30 min. Then the cells were collected and rinsed with PBS. They were mixed with 400 μL binding buffer, 5 μL Annexin V-FITC (KeyGEN BioTECH, Nanjing, China) for 15 min, and 10 μL PI for 5 min in the dark. Apoptosis was then assessed using flow cytometry (FACSAri3, BD Biosciences, USA) within 30 min.

### 2.15. NO Content Determination

The concentration of NO was determined using Griess reagent, which includes 1% sulfanilamide, 0.1% N-1-naphthylenediamine dihydrochloride, and 45% phosphoric acid, to detect NO in the culture medium. BV2 cells were seeded in a 24-well plate at a density of 2 × 10^5^ cells per well and co-treated with PF concentrations of 10^−6^, 10^−5^, or 10^−4^ M along with LPS (1 µg/mL) for 24 h, followed by exposure to 5 mM ATP for the final 30 min. Equal volumes of the supernatant and Griess reagent were mixed at room temperature, and the absorbance of the reaction mixture was measured at 540 nm using a spectrophotometer (HBS-1096A, Nanjing De Tie Biotechnology Co., Ltd., Nanjing, China).

### 2.16. Western Blot

Following euthanasia, hippocampal tissues from the mice were collected and lysed using RIPA buffer supplemented with a protease inhibitor (Roche Applied Science, Penzberg, Germany) under ice conditions. BV2 cells were seeded in a six-well plate at a density of 1 × 10^6^ cells. PF concentrations (10^−6^, 10^−5^, 10^−4^ M) along with LPS (1 µg/mL) were added to the cells for 24 h, followed by a 30-min exposure to 5 mM ATP. The supernatant was then removed, and the cells were lysed with RIPA buffer supplemented with a protease inhibitor, maintained on ice. The protein concentration was determined using a BCA protein assay kit (Thermo Scientific, Bend, OR, USA). The proteins were boiled in a buffer consisting of 62.5 mM Tris-HCl (pH 6.8), 2% sodium dodecyl sulfate, 20% glycerol, and 10% 2-mercaptoethanol. The proteins were separated by 10% SDS-PAGE and transferred onto a PVDF membrane. The PVDF membrane was blocked with 5% skim milk for 2 h and incubated overnight by various primary antibodies, including BDNF, TrkB, p-JNK, JNK, p-ERK, ERK, p-P38, P38, TLR4, p-NF-κB, NF-κB, NLRP3, Nrf2, HO-1, Keap-1, NQO-1, p-AKT, AKT, p-GSK-3β, GSK-3β, p-IκBα, IκBα, iNOS, and β-actin (1:1000, ABclonal, Wuhan, China). Then, the membrane was incubated with a secondary antibody (1:10,000, ABclonal, Wuhan, China). Finally, the bands were detected using an enhanced chemiluminescence method and analyzed using Image-J software (version 1.8.0 112, National Institutes of Health, Bethesda, MD, USA). The band density of the LPS (+) PF (−) group was set as 1.0.

### 2.17. Statistical Analysis

Analyses were conducted using the software of GraphPad Prism (GraphPad Prism 7.0, GraphPad Software, USA), with data presented as mean ± S.E.M. A one-way ANOVA was used, considering a *p*-value of less than 0.05 as significant.

## 3. Results

### 3.1. The Impact of PF on Depression-like Behavior in Mice

The SPT evaluates whether a mouse’s preference for sucrose water over regular water has decreased by comparing their consumption, which represents anhedonia, a core symptom of depression. The FST assesses negative behavioral patterns in response to stress by recording the time a mouse remains immobile (floating still) in an inescapable water environment, with this metric positively correlating with the severity of depression-like behavior. We also measured the weight of the mice and found no significant differences among the groups over 7 days. In the SPT, mice in the LPS group exhibited a significantly lower sucrose preference percentage compared to the Con group (*p* < 0.05, *p* < 0.01, Figure 1C), falling below 60%. After 7 days of PF treatment (20, 40, or 80 mg/kg/day), this reduction was significantly reversed (*p* < 0.01, Figure 1B). In addition, LPS significantly increased mice immobility time in contrast to the control group (*p* < 0.05, *p* < 0.01, Figure 1D). However, PF treatment (20, 40, or 80 mg/kg/day) significantly decreased immobility time, suggesting that PF alleviates despair in mice.

The OFT measures anxiety or depression levels by analyzing indicators such as the time spent in the central grid and preference for the edge area. The shorter the time spent in the central area is and the more frequent the activity at the edges is, the higher the levels of anxiety or depression. LPS markedly decreased the number of mice crossing the central grid, grooming, and standing in the OFT (*p* < 0.05, *p* < 0.01, Figure 1E–G). After 7 days of PF administration, the number of center crossing, grooming, and rearing events significantly increased in the PF group compared to the LPS group (*p* < 0.05, *p* < 0.01, Figure 1D–F).

### 3.2. The Impact of PF on LPS-Induced Neuronal Morphological Changes and the BDNF Pathway in C57 Mice

**Figure 2:**
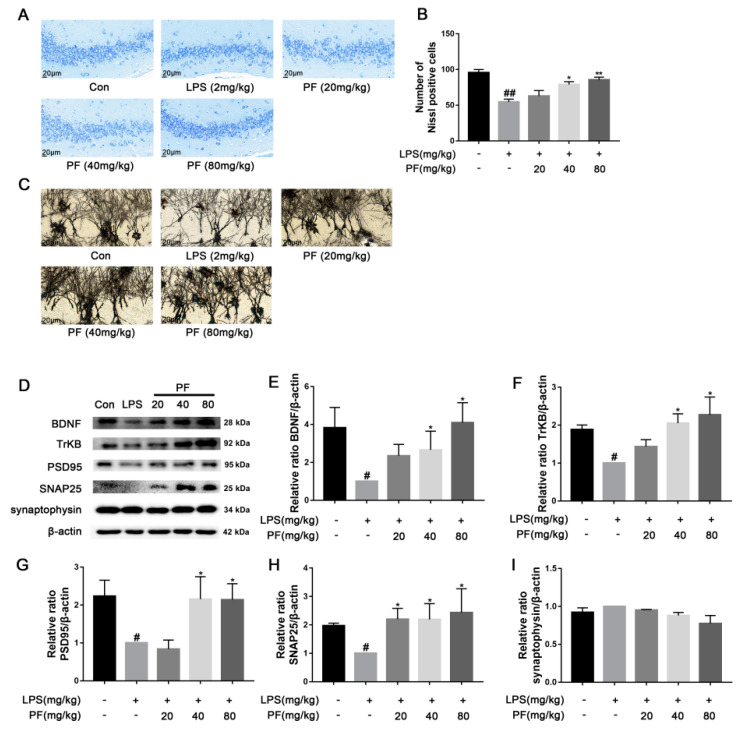
The impact of paeoniflorin (PF) on LPS-induced neuronal morphological changes based on representative images of neuronal dendrites visualized using Golgi-Cox and Nissl staining and BDNF pathway analysis in C57 mice. (**A**) Nissl staining of hippocampus CA3 region sections (magnification 400×, Scale bar = 20 μm). (**B**) Quantification of Nissl bodies in the hippocampus CA3 region. (**C**) Representative images of neuronal dendrites with Golgi-Cox staining (magnification 2500×, Scale bar = 5 μm). (**D**) Bands correspond to BDNF, TrkB, PSD95, SNAP25, synaptophysin, and β-actin. (**E**–**I**) The protein expression levels of BDNF, TrkB, PSD95, SNAP25, and synaptophysin relative to β-actin were assessed using quantitative analysis. All data are presented as mean ± SEM (n = 3). * *p* < 0.05, ** *p* < 0.01, compared to the LPS group. ^#^ *p* < 0.05, ^##^ *p* < 0.01, compared to the control group.

Significant evidence indicates that dysregulation of synaptic plasticity plays a role in the development of depression, leading to neuronal atrophy and weakened synapses in crucial brain areas like the hippocampus. Morphological changes were evaluated using Nissl and Golgi staining techniques. Synaptic plasticity is controlled by various signaling pathways, and disruptions in these pathways, such as the loss of neurotrophic factors, increase susceptibility to depression. BDNF is produced and released from postsynaptic spines, functioning in an autocrine manner to promote spine growth and density. To understand the mechanisms behind the antidepressant effects of PF, BDNF expression was analyzed using western blotting. PF significantly ameliorated the weakening of hippocampal synaptic plasticity in depressive model rats, as shown by a reduced number of pathologies including nerve cell crumpling, the abnormal deletion of Nissl bodies, disturbed Nissl body alignment (Figure 2A), increased dendritic spine density (Figure 2C), and increased BDNF, TrkB, PSD95, and SNAP25 protein levels (*p* < 0.05, *p* < 0.01 Figure 2D–I).

### 3.3. The Impact of PF on LPS-Induced Iba1, NLRP3, and DLG4 Protein Expression in C57 Mice

**Figure 3:**
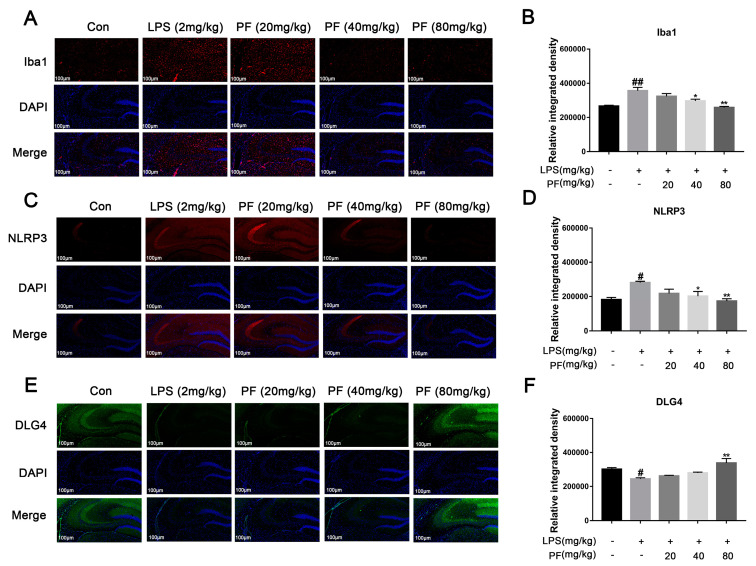
The impact of paeoniflorin (PF) on LPS-induced Iba1, NLRP3, and DLG4 protein expression in the hippocampus of C57 mice. (**A**,**B**) Iba1 protein expression (magnification 400×, Scale bar = 100 μm). (**C**,**D**) NLRP3 protein expression (magnification 400×, Scale bar = 100 μm). (**E**,**F**) DLG4 protein expression (magnification 400×, Scale bar = 100 μm). All data are presented as mean ± SEM (*n* = 3). Bar graph showing quantitative analysis of Iba1, NLRP3, and DLG4 based on quantified integrated optical density (IOD). * *p* < 0.05, ** *p* < 0.01, compared to the LPS group. ^#^ *p* < 0.05, ^##^ *p* < 0.01, compared to the control group.

Activated microglia can trigger neuroinflammation and release central cytokines, affecting synaptic plasticity. Iba1, NLRP3, and DLG4 represent microglial activation, inflammasome activity, and synaptic marker expression, respectively. PF significantly the upregulated the fluorescence intensity of the expression of synaptic plasticity-related key molecules of PSD95 and downregulated the fluorescence intensity of the expression of Iba1, thus increasing ameliorated microglial activation (Figure 3).

### 3.4. The Impact of PF on LPS-Induced Inflammatory Factors and Pathways in C57 Mice

**Figure 4:**
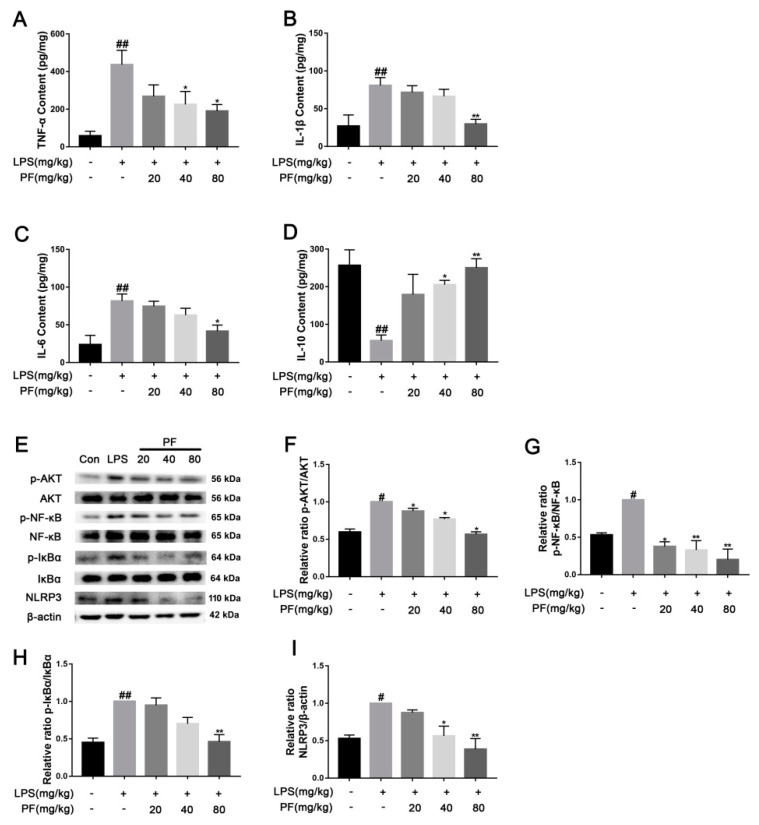
The impact of paeoniflorin (PF) on LPS-induced inflammatory factors and pathways in C57 mice. (**A**) TNF-α content, (**B**) IL-β content, (**C**) IL-6 content, and (**D**) IL-10 content in the serum. (**E**) Bands correspond to p-AKT/AKT, p-NF-κB/NF-κB, p-IκBα/IκBα, NLRP3, and β-actin. (**F**–**I**) Protein expression levels of p-AKT/AKT, p-NF-κB/NF-κB, p-IκBα/IκBα, and NLRP3/β-actin were subject to quantitative analysis. * *p* < 0.05, ** *p* < 0.01, compared to the LPS group. ^#^ *p* < 0.05, ^##^ *p* < 0.01, compared to the control group.

Elevated levels of cytokines such as TNF-α, IL-1β, and IL-6 may exacerbate neuroinflammatory responses, with their concentrations positively correlating with the severity of depression. These cytokines may also contribute to disease progression by affecting neural plasticity and synaptic function. In contrast, IL-10 alleviates neuroinflammatory responses by inhibiting the release of pro-inflammatory cytokines like TNF-α and IL-6, exhibiting antidepressant and neuroprotective effects. TNF-α, IL-1β, IL-6 and IL-10 cytokines were measured using ELISA. As depicted in Figure 4, TNF-α, IL-1β, and IL-6 levels were significantly elevated, while IL-10 levels were reduced in the mouse serum of the LPS group compared to that of the control group (*p* < 0.01). PF treatment mitigated these effects, lowering TNF-α, IL-1β, and IL-6 levels and enhancing IL-10 levels (*p* < 0.05, *p* < 0.01). This indicated that PF can ameliorate depressive-like behavior by regulating serum inflammatory cytokine levels in the LPS-treated model. Furthermore, in the resting state, IκBα binds to NF-κB and anchors it in the cytoplasm, preventing its nuclear translocation. When cells are stimulated by inflammation (such as LPS), IκBα is degraded, releasing NF-κB into the nucleus. AKT directly activates NF-κB through phosphorylation, enhancing its transcriptional activity and promoting the expression of inflammatory factors such as IL-6 and TNF-α. Additionally, NF-κB induces the synthesis of NLRP3 and pro-IL-1β. Upon activation of the NLRP3 inflammasome, it promotes the release of inflammatory factors like IL-1β. In this study, in Figure 4, AKT, NF-κB, and IκBα proteins exhibited a significantly higher levels of phosphorylation, and the NLRP3 levels were higher in the hippocampus in the LPS group compared to the control group (*p* < 0.01). PF treatment notably reduced these levels (*p* < 0.05, *p* < 0.01).

### 3.5. The Impact of PF on LPS-Induced Activation of the Keap1/Nrf2/HO-1 Pathway in C57 Mice

**Figure 5:**
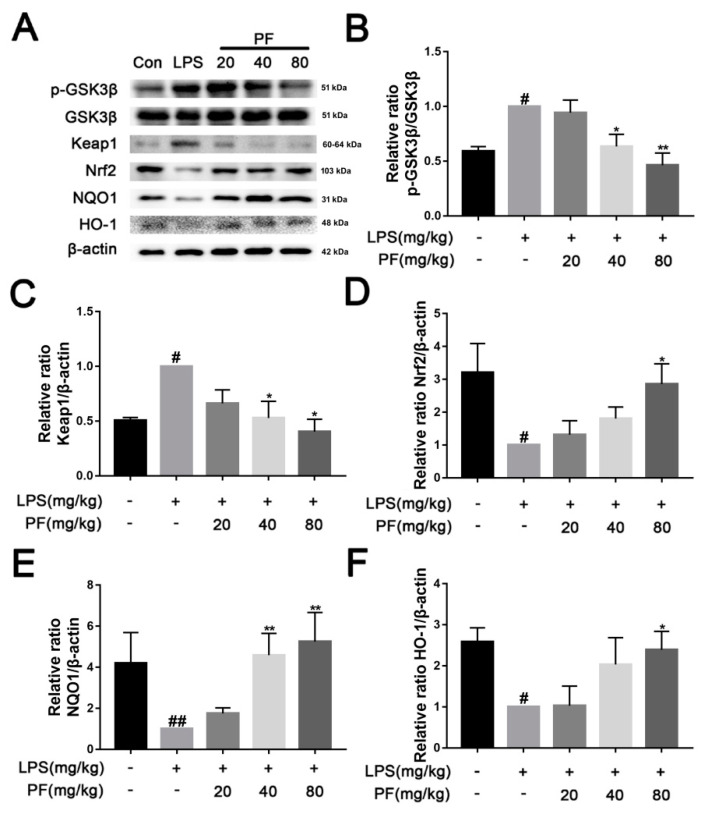
The impact of paeoniflorin (PF) on LPS induction of the Keap1/Nrf2/HO-1 pathway in the hippocampus of C57 mice. (**A**) Bands correspond to p-GSK3β/GSK3β, Keap1, Nrf2, NQO-1, HO-1, and β-actin. (**B**–**F**) Protein expression levels of p-GSK3β/GSK3β, Keap1, Nrf2, NQO-1, and HO-1 relative to β-actin were quantitatively analyzed. * *p* < 0.05, ** *p* < 0.01, compared to the LPS group. ^#^ *p* < 0.05, ^##^ *p* < 0.01, compared to the control group.

The Keap1/Nrf2/HO-1 pathway is a crucial defense system against oxidative stress. Under normal physiological conditions, Keap1 ubiquitinates and degrades Nrf2 to maintain its low activity level. However, when oxidative stress or inflammation occurs, Keap1 becomes inactive, allowing Nrf2 to accumulate and translocate to the nucleus, where it activates downstream antioxidant genes such as HO-1 and anti-inflammatory mediators, thereby restoring redox balance and inhibiting inflammatory responses. In Figure 5, LPS significantly reduced the levels of NQO1, Nrf2, and HO-1, while concurrently increasing the phosphorylation of GSK3β and the levels of Keap1 in the hippocampus (*p* < 0.05, *p* < 0.01). In contrast, PF significantly elevated the levels of NQO1, Nrf2, and HO-1 and concurrently decreased the phosphorylation of GSK3β and the levels of Keap1 (*p* < 0.05, *p* < 0.01).

### 3.6. The Impact of PF on BV2 Cell Viability and Oxidative Stress Induced by LPS

**Figure 6:**
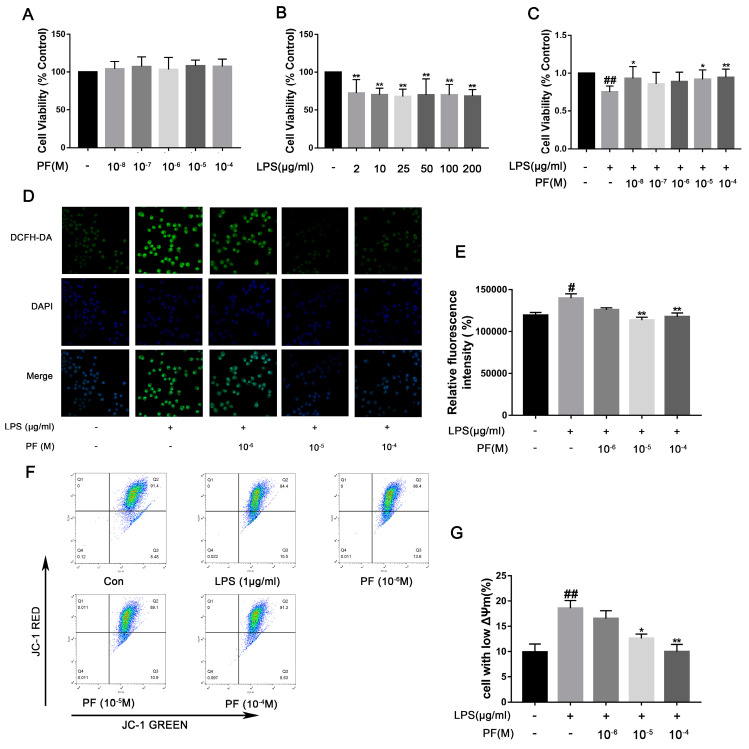
The impact of paeoniflorin (PF) on BV2 cell viability, ROS, and mitochondrial membrane potential induced by LPS. (**A**) Cytotoxicity of PF. (**B**) Viability of BV-2 cells after LPS treatment. (**C**) The impact of PF on BV2 cell viability in response to LPS treatment. (**D**) Representative fluorescence image of ROS. (**E**) Quantitative graph of average fluorescence intensity of DCFH-DA. (**F**) Flow cytometry of mitochondrial membrane potential. (**G**) The percentage of cells with low potential. Data are presented as mean ± SEM (*n* = 8). * *p* < 0.05, ** *p* < 0.01, compared to the LPS group. ^#^ *p* < 0.05, ^##^ *p* < 0.01, compared to the control group.

LPS activates NF-κB through a “priming signal”, while ATP acts as a “trigger signal” to activate the NLRP3 inflammasome, leading to the efficient secretion of factors such as IL-1β. Therefore, in this experiment, we used ATP co-treatment following LPS exposure in BV2 cells. Cells viability was evaluated using the CCK-8 assay. As depicted in Figure 6, PF concentrations of 10^−4^, 10^−5^, 10^−6^, 10^−7^, and 10^−8^ M did not impact BV2 cell viability. Exposure to LPS significantly decreased cell viability relative to the control group (*p* < 0.01). Conversely, 10^−5^ and 10^−4^ M PF treatment significantly enhanced cell viability compared to the LPS group (*p* < 0.05, *p* < 0.01, Figure 6), indicating that PF can counteract LPS-induced reductions in cell viability. However, 10^−6^ and 10^−7^ PF concentration do not show a statistically significant difference compared to the LPS group. This might be due to the low concentration of PF. LPS treatment markedly elevated intracellular ROS levels (*p* < 0.05, Figure 6D,E), whereas PF treatment substantially mitigated this increase (*p* < 0.01), suggesting that PF alleviated LPS-induced oxidative stress. Furthermore, flow cytometry analysis of JC-1 staining in Figure 6 showed that the proportion of cells with mitochondrial depolarization in the LPS group was 18.57 ± 1.53%. After treatment with different concentrations of PF (10^−6^, 10^−5^, or 10^−4^ M), the proportions were 16.57 ± 1.60%, 12.57 ± 0.90%, and 9.97 ± 1.40%, respectively. Analysis revealed that 10^−5^ and 10^−4^ M PF concentrations had statistically significant differences compared to the LPS group, showing that treatment with PF at concentrations of 10^−5^, and 10^−4^ M significantly reduced the number of cells with low potential (*p* < 0.05, *p* < 0.01).

### 3.7. The Impact of PF on BV2 Cell Apoptosis Induced by LPS

**Figure 7:**
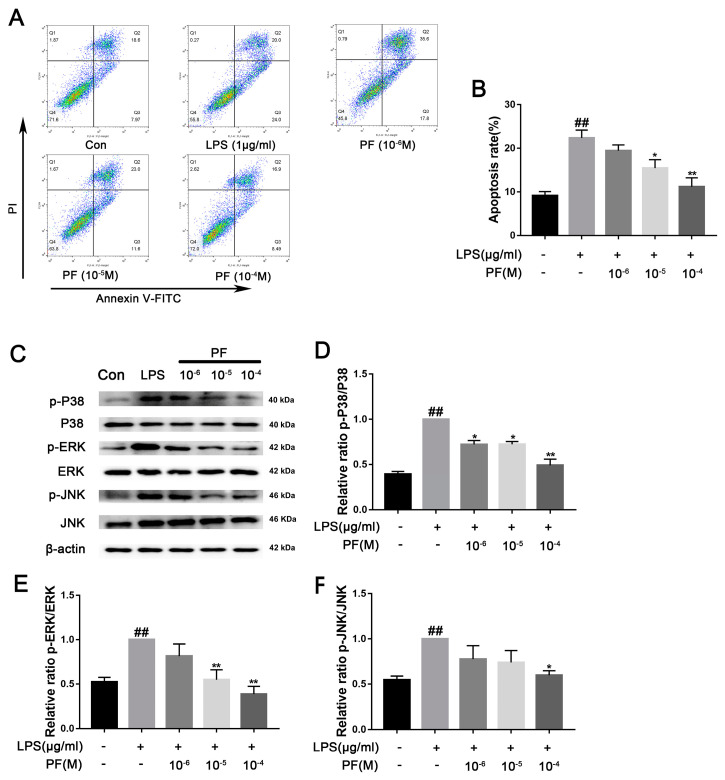
The impact of paeoniflorin (PF) on BV2 cell apoptosis induced by LPS. (**A**) Apoptosis flow cytometry. (**B**) Percentage of apoptotic cells. (**C**) Bands correspond to p-P38/P38, p-ERK/ERK, p-JNK/JNK, and β-actin. (**D**–**F**) Protein expression levels of p-P38/P38, p-ERK/ERK, and p-JNK/JNK were subject to quantitative analysis. Data are presented as mean ± SEM (n = 3). * *p* < 0.05, ** *p* < 0.01, compared to the LPS group. ^##^ *p* < 0.01, compared to the control group.

ROS can increase the activation of NLRP3 inflammasomes in microglia, thereby promoting the release of pro-inflammatory factors such as IL-1β, IL-6, and TNF-α. Additionally, the accumulation of ROS exacerbates mitochondrial membrane potential collapse and DNA damage, leading to neuronal apoptosis and microglial dysfunction. Lowering ROS levels can restore mitochondrial homeostasis and reduce the formation of neurotoxic microenvironments. In Figure 7, flow cytometry analysis of apoptosis showed that the proportion of apoptotic cells in the LPS group was 47.37 ± 3.07%. After treatment with different concentrations of PF (10^−6^, 10^−5^, or 10^−4^ M), the proportions were 50.87 ± 1.75%, 37.77 ± 1.59%, and 30.48 ± 2.95%, respectively. Analysis revealed that the concentrations of PF at 10^−5^ and 10^−4^ M had statistically significant differences compared to the LPS group. The apoptosis rate displayed a twofold increase in the LPS group relative to the control group (*p* < 0.05, *p* < 0.01). Furthermore, LPS exposure significantly increased the phosphorylation levels of P38, ERK, and JNK (*p* < 0.01). Conversely, PF at concentrations of 10^−6^, 10^−5^, and 10^−4^ M significantly reduced the levels of p-ERK, p-JNK, and p-P38 proteins in the control group compared to the LPS group (*p* < 0.05, *p* < 0.01, Figure 7).

### 3.8. The Impact of PF on BV2 Cell Inflammation Induced by LPS

**Figure 8:**
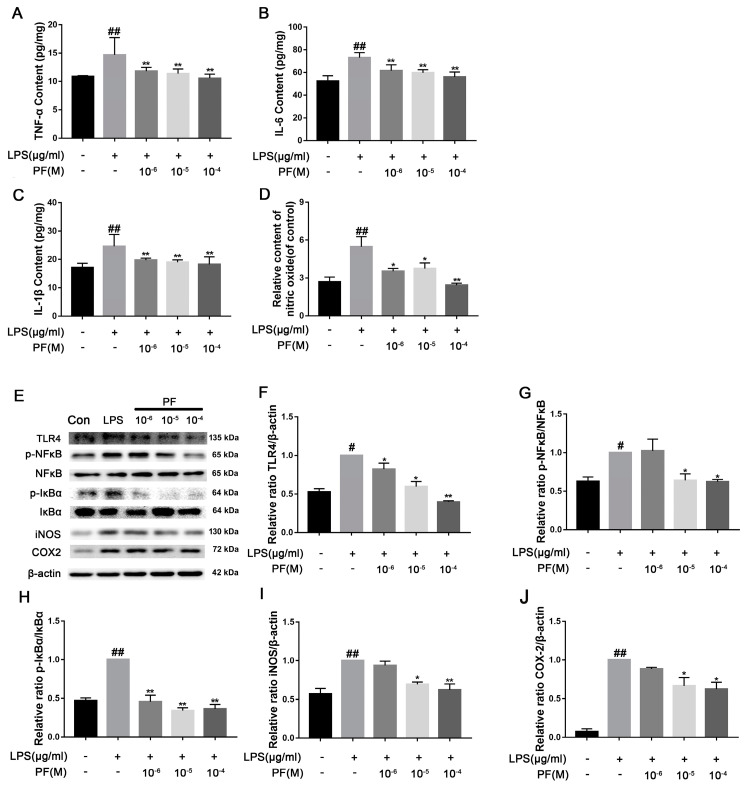
The impact of paeoniflorin (PF) on BV2 cell inflammation induced by LPS. (**A**) TNF-α content, (**B**) IL-β content, (**C**) IL-6 content, and (**D**) IL-10 content in BV2 cells induced by LPS. (**E**) Bands correspond to TLR4, p-NF-κB/NF-κB, p-IκBα/IκBα, iNOS, COX2, and β-actin. (**F**–**J**) Protein expression levels of TLR4, p-NF-κB/NF-κB, p-IκBα/IκBα, iNOS/β-actin, and COX2/β-actin were subject to quantitative analysis. Data are presented as mean ± SEM (n = 3). * *p* < 0.05, ** *p* < 0.01, compared to the LPS group. ^#^ *p* < 0.05, ^##^ *p* < 0.01, compared to the control group.

Treatment with LPS significantly elevated pro-inflammatory cytokine levels and NO concentrations in cells when compared to the control group (*p* < 0.01). Conversely, PF notably diminished TNF-α, IL-6, IL-1β, and NO levels (*p* < 0.05, *p* < 0.01, Figure 8A–D). Furthermore, LPS-treated BV2 cells exhibited significantly increased protein expression of p-NF-κB/NF-κB, p-IκBα/IκBα, COX2, and iNOS relative to the control group. PF effectively mitigated LPS-induced injury, resulting in a reduction in p-IκBα/IκBα (*p* < 0.05, *p* < 0.01, Figure 8E,H). In contrast, the expression of p-NF-κB/NF-κB, COX2, and iNOS proteins was significantly decreased at PF concentrations of 10^−5^ and 10^−4^ M. In addition, 10^−4^ M PF significantly decreased TLR4 protein expression (*p* < 0.05, *p* < 0.01, Figure 8E–I).

### 3.9. The Impact of PF on LPS-Induced Activation of the TLR4 Pathway 

**Figure 9:**
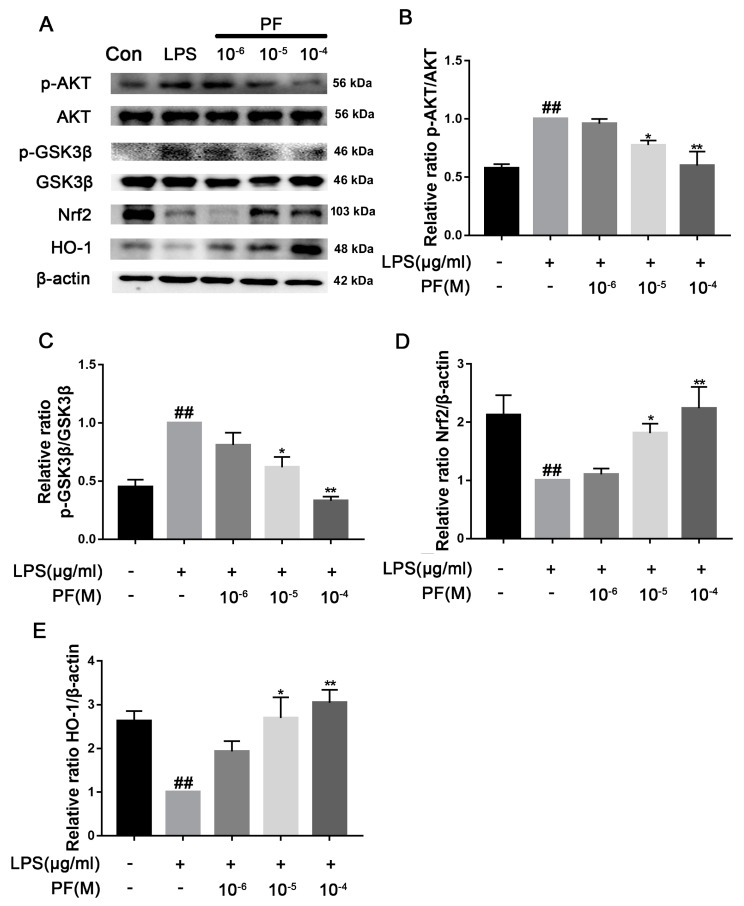
The impact of paeoniflorin (PF) on LPS-induced activation of the Nrf2/HO-1 pathway. (**A**) Bands correspond to p-AKT/AKT, p-GSK3β/GSK3, TLR4, Nrf2, HO-1, and β-actin. (**B**–**E**) Bands corresponding to p-AKT/AKT, p-GSK3β/GSK3β, Nrf2, and HO-1 relative to β-actin were subject to quantitative analysis. Data are presented as mean ± SEM (*n* = 3). * *p* < 0.05, ** *p* < 0.01, compared to the LPS group. ^##^ *p* < 0.01, compared to the control group.

There was a significant increase in TLR4, p-AKT/AKT, and p-GSK3β/GSK3β protein levels in the LPS group compared to the control group (*p* < 0.05, *p* < 0.01, Figure 9). In contrast, treatment with PF at concentrations of 10^−6^, 10^−5^, and 10^−4^ M resulted in a significant decrease in the protein expression of TLR4, p-AKT/AKT, and p-GSK3β/GSK3β in the control group relative to the LPS group (*p* < 0.01). Additionally, PF significantly upregulated the expression of Nrf2 and HO-1 proteins (*p* < 0.05, *p* < 0.01).

## 4. Discussion

In this study, we used an LPS-induced depression mouse model for the first time to explore the molecular mechanism of PF in the treatment of depression. Our results suggested that PF improved the LPS-induced depression-like behavior in mouse models of depression, inhibiting Keap1-mediated Nrf2 degradation and activation of the NLRP3 inflammasome. This prevented the increased levels of inflammatory factors in the mouse hippocampus and BV2 microglia in vitro. In addition, PF inhibited LPS-induced microglial activation and alleviated LPS-induced hippocampal neuron loss and synaptic plasticity damage. These results indicated that PF suppressed the activation of NLRP3 inflammasome in mice with LPS-induced depression-like behavior through the Keap1/ Nrf2/HO-1 pathway.

Animal models utilizing LPS induction or chronic stress are currently highly valuable for the screening of antidepressant drugs and the study of disease pathogenesis [16]. LPS is made up of a hydrophobic lipid, a hydrophilic core polysaccharide chain, and a hydrophilic O-antigenic polysaccharide side chain. Due to these features and its large size, LPS does not readily cross the blood-brain barrier (BBB) under normal physiological conditions. However, LPS can increase BBB permeability by triggering systemic inflammation. This makes it easier for other molecules (and potentially some LPS fragments) to enter the brain [17]. A single systemic injection of LPS causes behavioral changes that mimic acute systemic inflammation or infection, worsening depression [18]. Based on these facts, this study found that LPS can induce the occurrence of inflammatory reactions. After LPS treatment, NF-κ B is activated, pro-inflammatory cytokine release is induced, Iba-1 expression is increased, and ROS and NO concentrations are altered, leading to neuroinflammation in the brain, consistent with previous studies [19]. However, PF treatment significantly inhibited LPS-induced neuroinflammatory changes and significantly improved depression.

Depression and inflammation are interlinked, with each condition having the potential to precipitate the other. Individuals suffering from depression exhibit significantly elevated levels of inflammatory biomarkers [20]. The activation of the peripheral immune system elevates the levels of cytokines, which are subsequently transported to the central nervous system, thereby stimulating microglia. These activated microglia further enhance cytokine production. Importantly, during the onset of depression, microglia in brain regions associated with depression, such as the prefrontal and anterior cingulate cortex, exhibit significant activation [21]. Microglial activation can lead to synaptic defects and neuronal apoptosis, which is associated with the development of depression-like behavior. Inhibiting microglial activation can improve neuronal loss by reducing neuroinflammation, restoring synaptic integrity, and preserving the microglia-neuron network, which is crucial for neuronal health and function. Iba-1 staining showed that PF can inhibit LPS-induced hippocampal microglial activation. Nissl and Golgi staining revealed that PF reversed hippocampal neuronal loss and decreased dendritic spine density. Considering this role, PF may also indirectly affect astrocyte function by modulating the inflammatory environment.

Inflammation stimulates the production of ROS through various mechanisms, including immune cell activation, mitochondrial dysfunction, inflammatory mediator signaling, and enzymatic systems. Pro-inflammatory cytokines, such as TNF-α and IL-6, upregulate the expression of ROS-generating enzymes like NADPH oxidase by activating the NF-κB or MAPK signaling pathways. As a crucial signaling molecule, ROS trigger inflammatory responses within the central nervous system by activating redox-sensitive transcription factors, such as NF-κB [6]. Cells exposed to LPS can induce the production of intracellular ROS, and NF-κB is activated as an oxidative stress sensor [13]. Previous studies have found PF inhibits tributyltin chloride-induced apoptosis and glutamate-induced neurotoxicity via antioxidant mechanisms and Ca^2+^ antagonism [11]. In LPS-induced BV2 microglial cells, reduced cell viability, increased ROS, and a significant increase in cells with low mitochondrial membrane potential were observed. These features are associated with apoptosis and consistent with previous studies [22]. However, PF significantly ameliorates these cellular damages.

ROS and IL-1β are released after microglia activation, further aggravating neuronal damage. Oxidative stress activates PI3K/Akt signaling, which regulates pro-inflammatory protein expression [23]. However, the body’s defense system counters these redox changes. The Nrf2 response is essential for maintaining and restoring the in vivo balance after oxidative damage [6]. Keap1 binds and inhibits Nrf2 in the physiological state, and the LPS-induced accumulation of ROS can destroy the Keap1-Nrf2 complex and promote Nrf2 to enter the nucleus to activate the expression of antioxidant genes such as HO-1. HO-1 degrades heme to produce carbon monoxide (CO) and bilirubin, directly clears ROS, inhibits NF-κB phosphorylation, and blocks nuclear translocation and the release of pro-inflammatory factors (such as TNF-α and IL-6). Furthermore, Nrf2 activation inhibits NF-κB-dependent NLRP3 gene expression, reducing the synthesis of key proteins involved in inflammasome assembly [6]. Our study found that PF treatment significantly reduced Keap1 levels and increased Nrf2 and HO-1 expression, thus reducing NLRP3 expression. Additionally, PF treatment significantly decreased LPS-enhanced ROS accumulation, mitochondrial membrane potential, and inflammatory factors in BV2 microglia. These results fully suggested that PF can reduce oxidative stress through the Keap1/Nrf2/HO-1 pathway, thereby reducing LPS-induced neuroinflammation.

Increasing evidence suggests that excessive inflammation and oxidative stress are key events in the development of depression. The Keap1/Nrf2/HO-1 pathway plays a core regulatory role in LPS-induced inflammatory responses and depression. LPS-induced inflammation can lead to oxidative stress and neuronal damage, while the Keap1/Nrf2/HO-1 pathway integrates antioxidant and anti-inflammatory effects, serving as a common molecular hub linking inflammation and depression [6]. Nrf2-deficient mice exhibit increased cytokine production, while some Nrf2 activators show anti-inflammatory effects. Plant-derived and synthetic Nrf2 activators could be utilized as efficient anti-inflammatory drugs in neuropsychiatric disorders [24]. Nrf2 also inhibits microglial hyperactivation by suppressing p38 MAPK and the NF-κB signaling pathway [25]. Nrf2 agonists can reduce inflammatory responses in BV-2 cells [26]. Previous studies have shown that PF can reduce glutamate-induced neurotoxicity in PC12 cells via antioxidant mechanisms and alleviate cognitive impairment by inhibiting the inflammatory response though the NF-κB signaling pathway [11,27]. In our study, we found that PF alleviates LPS-induced neuroinflammation and depression through the Keap1/Nrf2/HO-1 signaling pathway. Regrettably, we did not assess the key proteins of the Keap1/Nrf2/HO-1 pathway using immunohistochemistry in brain tissue or by employing Nrf2 inhibitors or other compounds as tools to evaluate the impact of PF.

Depression significantly alters the number, density, and size of glial cells [28]. Microglia modulate neuroimmune pathways involving immune components like natural killer cells, cytokines, chemokines, Toll-like receptors, and growth factors. Activated microglia also trigger inflammasomes, which convert IL-1 β into its active form [29]. In this study, we noted marked microglial activation in the hippocampal CA3 region of depressed mice, resulting in pro-inflammatory cytokine production and NLRP3 inflammasome activation, which worsened depression. LPS binding to TLR4 recruits MyD88, triggering signaling pathways like NF-κB and MAPK [30]. The MAPK pathway activates NF-κB, which is crucial for expressing pro-inflammatory cytokines (e.g., TNF-α and interleukin), COX-2, and iNOS, leading to increased ROS and NO production [31,32]. Previous studies have shown PF inhibits activation of the IRAK1-NF-κB signaling pathway in peritoneal macrophages and attenuated bupivacaine-induced neurotoxicity in SH-SY5Y cells via suppression of the p38 MAPK pathway [33]. Similarly, research has found that PF inhibited atherosclerotic inflammatory cytokines IL-1β, IL-6, and TNF-α via the blockade of the TLR-4-mediated NF-κB signaling pathway [27]. In addition, PF reduced LPS-induced acute lung injury through inhibition of TNF-α and IL-1β secretion and upregulation of IL-10 production [34]. In our study, PF treatment downregulated LPS-induced TLR4 expression as well as p-P38, p-ERK, and p-JNK activation. It also inhibited the downstream NF-κ B signaling, thereby reducing cytotoxic factors such as COX2, iNOS, and TNF-α, improving the inflammatory response of BV2 cells and depressed mice.

LPS-induced inflammatory factors (such as IL-1β and TNF-α) inhibit the expression of key synaptic plasticity proteins such as BDNF and PSD-95, resulting in decreased dendrite spine density in the hippocampus and prefrontal cortex [35]. By activating TLR4, LPS triggers the activation of microglia and astrocytes, releases pro-inflammatory factors such as IL-1β and TNF-α, and activates the NLRP3 inflammasome to form a neuroinflammatory microenvironment that directly destroys synaptic function [36,37,38]. Furthermore, inflammatory factors (such as TNF-α) inhibit BDNF transcription and translation, while ROS reduces BDNF activity through oxidative stress, impacting neuronal survival and synaptic remodeling ability. Previous studies show that depressed patients have impaired neural plasticity and lower BDNF levels [9]. Our research also showed that LPS activates inflammasomes, lowers BDNF expression, and impairs neuroplasticity. PF treatment countered these effects by reversing inflammasome activation, reducing pro-inflammatory cytokines, increasing dendritic spine density, and upregulating synaptic proteins like SNAP25 and PSD95.

## 5. Conclusions

In summary, PF mitigates LPS-induced depression through NLRP3 suppression and Nrf2/HO-1 activation, offering a promising therapeutic strategy for inflammation-related depression-like behavior in mice.

## Data Availability

The data used to support the findings of this study are available from the corresponding author upon request.

## Abbreviations

BDNF: brain-derived neurotrophic factor; DLG4: recombinant discs, large homolog 4; FST: forced swim test; HO-1: heme oxygenase-1; Keap-1: Kelch-like ECH-associated protein 1; LPS: lipopolysaccharide; Nrf2: nuclear factor erythroid 2-related factor 2; NF-kB: nuclear factor-kappa B; OFT: open field test; PSD95: postsynaptic density protein 95; PF: paeoniflorin; ROS: reactive oxygen species; SPT: sucrose preference test; SNAP25: recombinant synaptosome-associated protein 25 kDa; TLR4: Toll-like receptor 4; TrkB: tropomyosin receptor kinase B.
